# Prediction of the Long-Term Effect of Iron on Methane Yield in an Anaerobic Membrane Bioreactor Using Bayesian Network Meta-Analysis

**DOI:** 10.3390/membranes11020100

**Published:** 2021-01-31

**Authors:** Dawei Yu, Yushuai Liang, Rathmalgodagei Thejani Nilusha, Tharindu Ritigala, Yuansong Wei

**Affiliations:** 1 State Key Joint Laboratory of Environmental Simulation and Pollution Control, Research Center for Eco-Environmental Sciences, Chinese Academy of Sciences, Beijing 100085, China; dwyu@rcees.ac.cn (D.Y.); lys15122117710@163.com (Y.L.); nthejani@yahoo.com (R.T.N.); tharindu_st@rcees.ac.cn (T.R.); 2 Department of Water Pollution Control Technology, Research Center for Eco-Environmental Sciences, Chinese Academy of Sciences, Beijing 100085, China; 3 University of Chinese Academy of Sciences, Beijing 100049, China; 4 BIOMATH, Department of Data Analysis and Mathematical Modelling, Ghent University, Coupure links 653, 9000 Ghent, Belgium

**Keywords:** methanogenic yield, ferric, anaerobic membrane reactor, Bayesian network meta-analysis, food processing wastewater

## Abstract

A method for predicting the long-term effects of ferric on methane production was developed in an anaerobic membrane bioreactor treating food processing wastewater to provide management tools for maximizing methane recovery using ferric based on a batch test. The results demonstrated the accuracy of the predictions for both batch and long-term continuous operations using a Bayesian network meta-analysis based on the Gompertz model. The prediction bias of methane production for batch and continuous operations was minimized, from 11~19% to less than 0.5%. A biochemical methane potential-based Bayesian network meta-analysis suggested a maximum 2.55% ± 0.42% enhancement for Fe2.25. An anaerobic membrane bioreactor improved the methane yield by 2.27% and loading rate by 4.57% for Fe2.25, operating in the sequenced batch mode. The method allowed for a predictable methane yield enhancement based on the biochemical methane potential. Ferric enhanced the biochemical methane potential in batch tests and the methane yield in a continuously operated reactor by a maximum of 8.20% and 7.61% for Fe2.25, respectively. Copper demonstrated a higher methane (18.91%) and sludge yield (17.22%) in batch but faded in the continuous operation (0.32% of methane yield). The enhancement was primarily due to changing the kinetic patterns for the last period, i.e., increasing the second methane production peak (k_71_), bringing forward the second peak (λ_7_, λ_8_), and prolonging the second period (k_62_). The dual exponential function demonstrated a better fit in the last three stages (after the first peak), which implied that syntrophic methanogenesis with a ferric shuttle played a primary role in the last three methane production periods, in which long-term effects were sustained, as the Bayesian network meta-analysis predicted.

## 1. Introduction

Maximizing the recovery of biogenic methane in the agriculture sector through process-based studies is needed [1]. To this end, methanogenic kinetics based on biochemical methane potential may be a promising method to progress from batch to long-term continuous methane recovery. The Biochemical Methane Potential (BMP) test is a method to determine the feasibility of an agent to stimulate methane production in anaerobic digestion. The test provides the precise kinetics of methane production under well-controlled conditions [2]. These conditions can involve different substrates, agents, and dosages [3,4]. Therefore, the stimulation can be evaluated as the percentage of methane increase as compared to a control group. For example, 5~350-mmol magnetite stimulates a 1.6%~16.1% biogas production potential (*G_0_*, mL/gVS_added_) in BMP batch tests [5], while nano-magnetite 5~350 mmol stimulates a −6.9%~6.0% biomethane production potential (*G_0_*, mL/gVS_added_) in batch tests [6]. A similar percentage enhancement is expected in continuous reactors in a batch test. However, the enhanced rates are different in a batch and a continuous reactor [7]. For example, 20-mmol magnetite (100–700 nm particle size) simulates a 4.5% methane yield in continuous operations [8], even higher than a similar dosage in a batch test [5,6]. Ferric could trigger multiple effects in anaerobic digestion, e.g., trace elements of methanogens, activator or function center of metalloenzyme (F420), flocculation, and enhance the direct interspecies electron transfer (DIET) by different mechanisms [9]. These effects could demonstrate different impacts on the short-term and long-term effects. Comparably, copper also impacted the methane yield but demonstrated limited mechanisms in anaerobic digestion, e.g., copper works as a trace element for methanogens [10]. Therefore, the short-term effects were more similar with the long-term effects for copper [11]. The differences (of ferric supplement) between the batch and continuous reactors highlight the needs for an evaluation method, especially for ferric supplements, which demonstrates the coexistence of multiple mechanisms [12]. The copper supplement demonstrated limited mechanisms and similar iron strength, which makes copper an ideal control treatment for developing the prediction method. Such an evaluation method should predict the enhancement effects in a continuous reactor based on a corresponding batch test. However, there is still a disparity between the methods used for the batch and continuous reactors.

There are also attempts to expand the biochemical methane potential to a long-term methane recovery to evaluate its biodegradability and methane yield. The biochemical methane potential in anaerobic digestion was expected to be the five-day or 20-day biochemical oxygen demand in an active-sludge treatment. The first step is the early prediction of methane production, which can be realized by modeling methane production in the initial days [12,13]. An early prediction of the biochemical methane potential can be achieved through the static and kinetic modeling of the initial gas production [13]. A fifty-day total biogas production can be predicted based on 14 days in an anaerobic biodegradation test (BMc) using the same kinetic modeling. The next step is the extension of batch testing of continuous reactors; for example, connecting the biochemical methane potential to the methane yield. A full biochemical methane potential implies an infinite digestion time (also known as the retention time), which is the significant difference from a practical reactor. A reactors’ hydraulic retention time can range from hours to 20~30 days for anaerobic reactors, and 5–40 h for aerobic reactors. Five days is long enough for an anaerobic reactor, but for BMP(*t*), it is difficult to choose an acceptable time that balances being efficient and useful [14]. Even if *t* is set as the reactor-specific retention time, the BMP(*t*) is too large, as compared with the reactor’s methane yield. Model predictions based on the components have also been studied [3], but biomass components change a lot between types and seasons; thus, they rely on a chemical components analysis, which is the main limitation.

In general, there are three types of kinetic models for predicting methane production. The Monod models were implemented as a series of models from the International Water Association, e.g., anaerobic digestion model No.1 and active-sludge models [15]. The Monod quadratic (MQ) model contains a modified Monod model with a similar mathematic form as the Michaelis–Menten model. The model can plot curves for balanced growth or the competition of substrates in a simple form [16]. The first-order models include the logarithmic growth phase in anaerobic digestion. The first-order models are applicable in scenarios in which there is a single, limited step [17]. The limit is usually the consumption of substrates. Gompertz models are typical “S”-style curve equations [13]. The Gompertz model can shape two methane production peaks and a lag time, which has attracted a lot of intention as regards inhibition and stimulation in relation to methanogenic kinetics [18].

In this context, the objective of this study was to statistically predict the long-term effects of ferric on methane production and was developed in an anaerobic membrane bioreactor treating food processing wastewater, based on the biochemical methane potential from the batch results using the Bayesian network meta-analysis. The method developed allows for a better understanding of the long-term kinetic effects and dosage optimization of ferric, where copper was the control treatment.

## 2. Material and Methods

### 2.1. Scheme and Operation of the Anaerobic Membrane Bioreactor

A lab-scale anaerobic membrane bioreactor (AnMBR) was set up for testing the long-term effects of ferric on methane production in an AnMBR treating starch wastewater. The working volumes of the acidogenesis phase or methanogenesis phase were 2 L and 4 L, respectively. The temperature was maintained at mesophilic conditions 37 ± 2 °C and 37 ± 0.3 °C by electrical heating and water bath, respectively. The methanogenesis phases were equipped with an online pH sensor (Hach Inc., Loveland, CO, USA) and biogas flow rate meter (μFlow, Bioprocesses AB., Lund, Sweden) for process monitoring. A tubular membrane (PVDF, 0.01 m^2^, 200,000 Da, Berghof GmbH, Eningen, Germany) was used for the effluent, externally driven by a centrifugal pump (OW-370A, Taizhou Qibo Ltd., Shandong, China) to create crossflow (3 m⋅s^−1^) for fouling mitigation. More details about the reactor can be found in [19]. Before the acidogenesis phase, the influent was injected using a peristaltic pump (100, Lange Ltd., Heibei, China), which was measured using an online electronic balance (TD10000, 0~10,000 ± 0.1 g, Tianjin Tianma Ltd., Tianjin, China).

The influent was obtained from a potato starch factory in Hebei Province, one of the top five private starch enterprises in China. The influent was filtered using a 0.15-mm screen, and the chemical oxygen demand (COD) and suspended solid (SS) were 27.61 ± 3.49 g⋅L^−1^ and 6.21 ± 0.84 g⋅L^−1^, respectively. The reactor was inoculated with anaerobic sludge from an egg-shaped digester, Xiaohongmen Wastewater Treatment Plant, Beijing, China [20].

**Figure 1:**
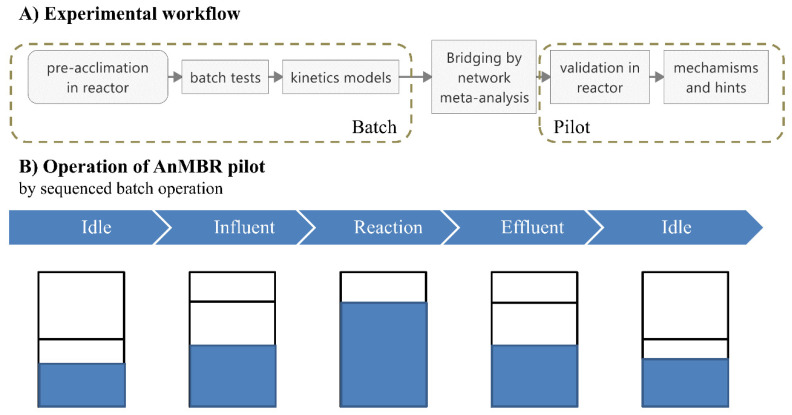
(**A**) Workflow of the prediction. (**B**) Sequenced batch operation of the semicontinuous anaerobic membrane bioreactor.

The AnMBR automatically operated as an anaerobic sequencing batch membrane reactor [21], where similar kinetic characteristics can be expected as those in the kinetic model. The automation strategy (Figure 1) was implemented on a BIO-5GCA lab fermenter (Shanghai Bailun Bio-Technology Co., Ltd., Shanghai, China). After the initial idle stage, the influent stage was performed, in which the AnMBR was fed with starch wastewater until the high water-level was met or the pH dropped. During the reaction stage, mechanical stirring (120 ± 4 rpm) was applied to achieve a completely mixed condition. The effluent stage began at the end of methane production and continued until the pH or water level dropped beyond a certain threshold [19]. After the effluent stage, another cycle followed. The augmentation was implemented in three stages: sCtrl (without dosage), sFe (dosed with ferric), and sCu (dosed with copper). The first stage, i.e., sCtrl, was characterized by a constant organic loading rate (ORL) to achieve stable operations [19]. The other stages used ferric chloride and copper chloride at optimized dosages, respectively.

### 2.2. Apparent Kinetics of Methanogenesis

The biochemical methane potential batch tests were performed to investigate the effects of ferric and copper on the methane production kinetics in three parallels (Figure 1A). The effluent of the acidogenesis phase was made up of substrates. The mixed liquor from the methanogenesis phase was the inoculum for the methanogenesis kinetic tests and modeling. The experiment was performed using the Automatic Methane Potential Test System (AMPTS-II, Bioprocess Control AB., Lund, Sweden) per the instrument’s manual. The ferric and copper were ferric chloride and copper chloride, respectively. The methane production kinetic parameters were estimated according to [13]. The models below are as follows: Equation (1)—first-order model, Equation (2)—a first-order rate model with a variable order of time dependency, Equation (3)—a combination of two first-order rate models, Equation (4)—a Monod-type model, Equation (5)—a quadratic Monod model, Equation (6)—a modified Gompertz model, Equation (7)—a Gompertz model, and Equation (8)—a logistic function model. The Gompertz and Logistic models were added to estimate the lag phase parameter [12,18,22]. These models were calibrated with cumulative methane production data from the batch tests. The relative root mean square error (rRMSE) and R^2^ were calculated according to [13]. The residuals were calculated according to [12].

(1) $$\mathrm{BMP}\left(t\right)=B_{1}\cdot \left(1-exp\left(-k_{1}\cdot t\right)\right)$$

(2) $$\mathrm{BMP}\left(t\right)=B_{2}\cdot \left(1-exp\left(-k_{2}\cdot t^{\gamma }\right)\right)$$

(3) $$\mathrm{BMP}\left(t\right)=B_{3}\cdot \left(1-X\cdot exp\left(-k_{31}\cdot t\right)-\left(1-X\right)\cdot exp\left(-k_{32}\cdot t\right)\right)$$

(4) $$\mathrm{BMP}\left(t\right)=B_{4}\cdot \left(\frac{k_{4}\cdot t}{1+k_{4}\cdot t}\right)$$

(5) $$\mathrm{BMP}\left(t\right)=B_{5}\cdot \left(\frac{t^{2}}{t^{2}+k_{51}\cdot t+k_{52}}\right)$$

(6) $$\mathrm{BMP}\left(t\right)=B_{6}\cdot exp\left(-\frac{\theta_{1}\cdot exp\left(-k_{61}\cdot t\right)}{k_{61}}-\frac{\theta_{2}\cdot exp\left(-k_{62}\cdot t\right)}{k_{62}}\right)$$

(7) $$\mathrm{BMP}\left(t\right)=B_{7}\cdot exp\left(-exp\left(k_{71}\left(\lambda_{7}-t\right)+1\right)\right)$$

(8) $$\mathrm{BMP}\left(t\right)=B_{8}\cdot \left(\frac{1}{1+exp\left(k_{81}\left(\lambda_{8}-t\right)+2\right)}\right)$$

Here, BMP(*t*,), *B_n_*, and *k_n_* refer to the cumulative methane production(at time *t*), BMP_∞_ (biochemical methane potential, NmlCH_4_/gCOD), and the rate constants for different kinetic models (*n*), respectively. Nomenclature of parameters are listed in Table S1. The BMP_∞_ was used for the following analysis in the Bayesian network meta-analysis.

The dosages of the ferric and copper additions are presented in Table S2. Each of the dosages was analyzed as a treatment. The dosage was designed to also consider the side effects of ferric chloride as a flocculating agent. The upper limit of the discharging standard of trace elements/heavy metals was Cu ≤ 2.0 mg⋅L^−1^.

### 2.3. Effects Evaluation by Bayesian Network Meta-Analysis

A network meta-analysis allows for the comparison of multiple treatments using estimates of rank probabilities based on the Bayesian approach [23]. The network meta-analysis is a widely used statistical method in evidence-based medicine, which is used for evaluating the effects of treatments with multiple mechanisms. The hypothesis was that higher dosages have comparatively more effects if considering the tested enhancement rate as a posterior probability of the enhancement happening in each dosage condition. The Bayesian network meta-analysis (BNMA) allows for decoupling of the multiple effects from a higher dosage to a lower dosage. The BNMA is an extension of the classical pairwise meta-analysis. The BNMA allows to compare multiple interventions based on both head-to-head comparisons within treatments and indirect comparisons across treatments. The BNMA method was used to predict the methane yield of the reactor with the BMP_∞_ results from the batch test. The BNMA was performed using the BMP_∞_ as the input. The Bayesian network meta-analysis (BNMA) model for the comparison of multiple treatments was performed based the central ideal of Bayes’ Theorem, which incorporates subjective prior knowledge into statistical inference (Equation (9)). The theorem allows for an estimation of the probability of an effect of event A (daily methane productions at a higher Ferric dose A) based on another event, B (daily methane productions at a lower Ferric dose B).

*P*(*A*|*B*) ∝ *P*(*B*|*A*) × *P*(*A*) (9)

The *P(A)* is what we want to estimate. To estimate the *P*(*A*), which represents the distributions of daily methane production, we denoted a set of parameters *θ* that characterized the distribution. Instead of B (true daily methane production at a lower Ferric dose B), here, we only collected actual experimental data (with errors, explained in the next paragraph) through which we estimated θ. The observed daily methane production was store in vector Y, which followed the distribution *P*(Y).

*P*(*θ*|Y) ∝ *P*(Y|*θ*) × *P*(*θ*) (10)

A conventional meta-analysis is defined as a pairwise meta-analysis, which is equivalent with a multilevel random effects model. The model works under the so-called assumption of exchangeability, where we not only assume that effects of individual treatments deviate from the true intervention effect of all treatments due to sampling error but that there are other sources of variance introduced by the fact that the studies do not stem from on single bacteria but from microbial communities. Therefore, there is not only one true effect size of daily methane production but a distribution of true effect sizes, and we want to estimate the mean of this distribution of true effect sizes. The model assumes that, when the observed effect size (observed daily methane production) k_n_’ of an individual dose A deviates from the true effect size k_T_ (true increase of daily methane production caused by the dose A), the reason for this is that the estimate is burdened by (sampling) error ϵ_k_ and an extra/second source of error ζ_k_. The second source of error is introduced by the fact that even the true effect size k_T_ is only part of an overarching distribution of true effect size with the mean μ (Equation (11)).

k_n_’ = k_T_ + μ + ζ_k_ (11)

To estimate the variance of the distribution of true effect sizes (τ^2^), several estimators (of τ^2^) are implemented in *meta*. For a conventional pairwise meta-analysis, we included *K* studies and collected the effect size *Y*_k_ for each of our studies k = 1, …, 5. We defined the fixed-effect model as Equation (12):

*Y*_k_∼*N*(k_T_, S_K_^2^) (12)

where we calculate the likelihood (the *P*(Y| *θ*) part from above) of our effect sizes, assuming that they follow a normal distribution drawing from the distribution of the one true effect size k_T_, which has the variance s_k_^2^. We do not assume that each study is an estimator of the one true effect sized k_T_ but that there are study-specific true effects k_n_ estimated by each effect size Y_k_. Furthermore, these study-specific true effects are again an overarching distribution of true effect sizes, which is defined by its mean *d* and variance τ^2^, our between-study heterogeneity.

k_n_∼*N*(*d*, τ^2^) (13)

In the Bayesian model, we also give an uninformative prior distribution to both *d* and τ^2^. The Bayesian network meta-analysis was therefore formulated by the Bayesian meta-analysis model for a pairwise meta-analysis. The Bayesian network meta-analysis can consist of different treatments being compared and denote a single effect size (daily methane production) obtained from one (experimental observation of) treatment/dose *a* that was compared to treatment/dose *b* as Equations (14) and (15). The study/inoculum-specific true effect for the comparison of *a* and *b* (ferric dose 1 to 5) is assumed to be part of an overarching distribution of true effect sizes with mean *d_ab_*, where *d_1a_* is the effect of treatment *a* compared to the predefined reference treatment without ferric supplement [24].

*Y*_kab_∼*N*(k_Tab_, s_k_^2^) (14)

k_nab_∼*N*(*d_ab_*, τ^2^) (15)

The Bayesian network meta-analysis is therefore performed using the *gemtc* and *rjags* package in R 3.7.0 (R Foundation, New York, USA) with the existence of a software called JAGS (Just Another Gibbs Sampler) [24]. The Network graph, *mcmc1* and *mcmc2*, and *nodesplit* plot and forest() function were plotted herein for the analysis.

### 2.4. Microbial Community Analysis

Mixed liquor of 0.4 g from the methanogenesis phase, where ferric or copper was added, was collected for DNA extraction with the QIAamp DNA Stool Mini Kit (Qiagen, Germantown, MD, USA). The reactor’s mixed liquor was sampled at the end of each stage for DNA extraction. These DNA extracts were marked as Ctrl, Fe, and Cu. PCR amplicons were purified with a DNA purification kit (BioFlux, Japan). The quality and concentration of the extracted DNA were determined through 1% agarose gel electrophoresis and NanoDrop ND-1000 (NanoDrop, Wilmington, DE, USA), respectively.

The microbial community of the samples was determined through a high-throughput sequencing method. The PCR primers 515F and 806R were selected for the 16S V4 region. DNA was amplified following the protocol, as previously described. The amplicons were sent out to Novogene Co., Ltd. in Beijing for small-fragment library construction and pair-end sequencing using an Illumina Mi-Seq sequencing system (Illumina, San Diego, CA, USA). Sequencing reads were assigned to each sample based on a unique barcode. Pairs of reads from the original DNA fragments were merged using FLASH [25] and filtered out using UCHIME [26]. Barcode and primers were removed from sequencing reads. After the above filtration, the minimum sequencing depth was 30,578 clean reads. Normalization of the sequence number was conducted for 30,578 sequences to make a fair comparison with the same sequencing depth. The sequences were deposited in MG-RAST under project mgp17379 [27].

The taxonomic classification of the sequences in each sample was conducted individually using the Ribosomal Database Project (RDP) Classifier, as previously suggested in [28]. The sequences in different taxonomy levels were assigned at the bootstrap cut-off of 50%, as suggested in [29].

### 2.5. Physicochemical Analysis

Chemical oxygen demand (COD), total solids (TS), volatile solids (VS), and ammonia were determined using the APHA method. Total carbon (TC), total organic carbon (TOC), and total inorganic carbon (TIC) were determined with a liquiTOC II (Elementar Analysensysteme GmbH, Germany). Online sensors of Oxidation–Reduction Potential (Hach Inc., Loveland, CO, USA) and electrical conductivity (EC) (Hach Inc., Loveland, CO, USA) were equipped, and the data were acquired using the programmable logic controller (PLC, S7-200, Siemens Inc., Munich, Germany) in the fermenter.

Sludge and bacteria morphologies were observed with electronic scanning microscopy with an electronic microscopy FE-SEM (Hitachi S-4160, accelerating voltage of 15 kV, Hitachi, Tokyo, Japan) in dry mode (10 Torr, dry biomass). Biogas yield and specific methanogenic activity were calculated as suggested in [30].

### 2.6. Statistical Analysis

The paired-samples *t*-test was used to evaluate the significance of the differences in the methane production caused by the addition of ferric and copper during the anaerobic digestion of starch wastewater. Pearson correlations were used to assess the association between kinetics parameters, and a *p*-value < 0.05 was considered to be statistically significant. The methanogenic kinetics were calibrated with the *CurveFit.jl* and *Optim.jl* packages in Julia 1.3.1 (Julia Lab, Massachu-setts Institute of Technology, Cambridge, UK). A dendrogram was plotted using an average link between groups to identify the dominant kinetic patterns. The Bayesian network meta-analysis was performed with the *bnma* package in R 3.7.0 (R Foundation, New York, USA) based on JAGS (Just Another Gibbs Sampler 4.3.0). The evolution of the microbial community was compared in pairs among sCtrl, sFe, and sCu by a two-sided Welch’s *t*-test in STAMP, in which it was filtered by *p* > 0.01 to decipher the microbial community response to ferric after the long-term augmentation of ferric in the anaerobic membrane bioreactor treating starch wastewater.

## 3. Results

### 3.1. Enhancement of Cumulative Methane Production in the Batch Test

**Figure 2:**
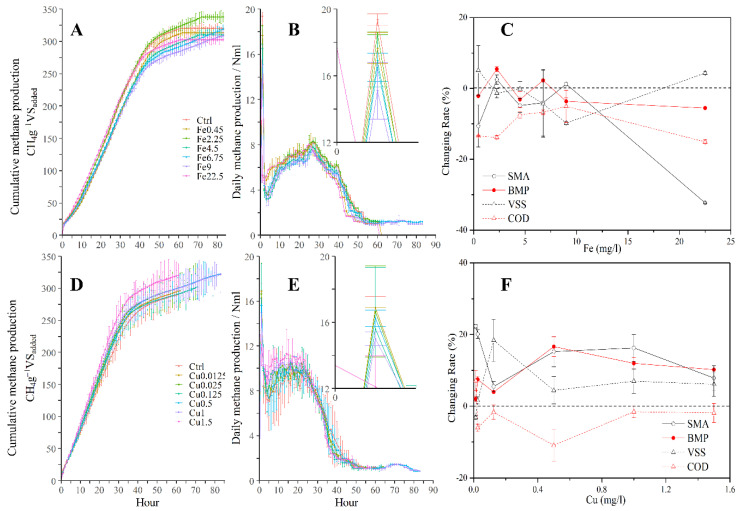
The profiles of cumulative methane production, daily methane production rate, and performance changes for ferric ((**A**), (**B**), (**C**)) and copper ((**D**), (**E**), (**F**)) during the Biochemical Methane Potential (BMP) batch test, respectively. The error bar was the standard error of average in three parallel tests. The dosage (mg/L) is listed after the elements (Fe and Cu) in the legend. The first peak of the methane production rate is amplified in the subfigures, respectively. The SMA, BMP, VSS, and COD refer to specific methanogenic activity, biochemical methane potential, violate suspended solid, and chemical oxygen demand, respectively. The changing rate was calculated by comparing the augmented experimental group with the control group, where the COD removal demonstrated a stable improvement in all ferric groups.

The paired-samples *t*-test indicated that ferric and copper significantly influenced the methane production in the batch anaerobic digestion of starch wastewater (*p* < 0.05). The ferric improved the cumulative methane production by a maximum of 8.20% at Fe2.25 and further increased during ferric-reduced methane production (Figure 2A). There were two methane production peak periods (hour one and hour 27) for the daily methane production. The first peak was generally the same among treatments, while the improvement of the methane production mainly happened later during the second peak (hours 25–30). The easily degradable organics formed the first peak, and the poorly biodegradable organics formed the second peak. This is easy to understand, because starch wastewater is a mixture of organic compounds with different biodegradability. This indicates that ferric can enhance the degradation of poorly biodegradable organics. Copper improved the cumulative methane production by a maximum of 7.9% at Cu1.5 and increased the biochemical methane potential (BMP) by a maximum of 18.91% at Cu0.5. The copper improved more in the first methane production peak than the second peak. The violate suspended solid (VSS) and specific methanogenic activity (SMA) were increased more, implying a higher growth of sludge. The increased sludge may be helpful for other types of anaerobic reactors but a burden for the anaerobic membrane bioreactor where extra sludge treatment is needed. A lower COD removal rate was achieved with copper than the ferric supplement. A lower COD removal rate implied more effluent organic matters and foulants, which is also a weakness for an anaerobic membrane bioreactor. Therefore, copper was less noticed on further investigation.

The methane yield is another important factor in a continuous reactor, because the digestion time is the essential operational parameters for continuously operated reactors (Figure 2). There is a maximal daily methane production (mL, Figure 2B), a maximum methane production rate (R_max_, mL·g^−1^VS_added_·d^−1^, M7 Gompertz model), and a specific methanogenic activity (SMA, gCOD·gVS^−1^·d^−1^, Figure 2C), which are used to describe the methane production rate. The maximum daily methane production is represented as the first methane production peak in which ferric is reduced between the two extremes by −4.13% and −21.96% for Fe2.25 and Fe9, respectively. Ferric was able improve the maximum methane production rate by a maximum of 7.70% for Fe22.5, with decreasing amounts of ferric reducing the maximum methane production rate to varying degrees. The specific methanogenic activity changed most significantly with ferric dosages in the batch (Figure 2C). Ferric was able to improve the specific methanogenic activity by a maximum of 2.5% for Fe2.25, higher than copper (Figure 2D). A further increase in ferric reduced the specific methanogenic activity by a maximum of −32.3% for Fe22.5. This indicates that ferric was able to accelerate the degradation speed, especially for poorly biodegradable organics. Ferric improved the COD removal by a maximum of 13.8% for Fe2.25. The ferric groups demonstrated a higher average COD removal than the copper group (Figure 2E,F). The COD will be converted to biogas (methane and CO_2_), microorganisms, and inert compounds in a long-term operation. The conversion of poorly biodegradable organics was enhanced by ferric. This further indicates that ferric was able to enhance the degradation of the poorly biodegradable organics.

### 3.2. Effect of Dosages on the Kinetics Parameters in the Batch Test

The methane production rates were also fitted as the kinetics parameters in the multiple kinetic models (M1~M8) with the Levenberg–Marquardt algorithm. While the cumulative methane productions were plotted using B (BMP(*t*) and B_n_), the methane production rates were depicted using the kinetic parameters (*k_n_, γ, λ,* etc.). The kinetics of methane production mainly include two groups of critical characteristics. The first group is made up of speed parameters: *k_n_* as the methane production rate constant, *X*, and *θ_n_* as the correction coefficient for the rate constant. The second group is made up of time dependency parameters: *γ* as the modified time dependency, and *λ* as the lag time. This is ferric evolving in multiple electron processes at various speeds.

**Figure 3:**
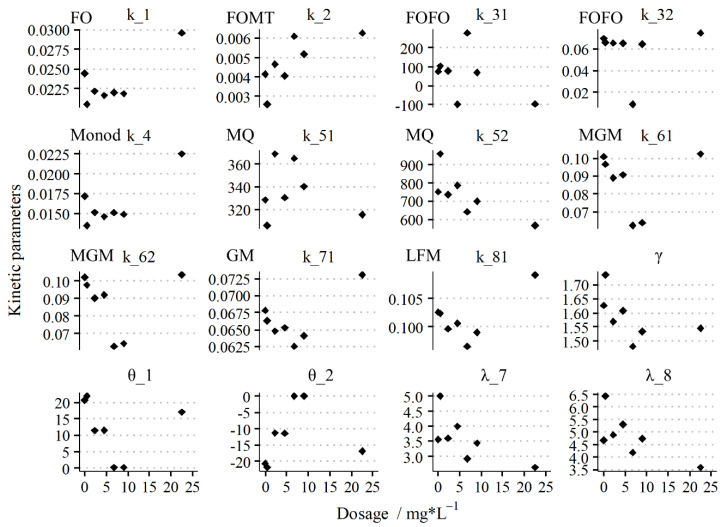
Facet plot of the rate parameters of different methanogenic kinetics under different ferric dosages. The results clearly demonstrate that the daily methane production rate improved with ferric augmentation. The nomenclature is presented in Table S1.

Figure 3 shows the ranges of the kinetic parameters of different models at ferric dosages from 0 mg⋅L^−1^ to 22.5 mg⋅L^−1^, in which 0 mg⋅L^−1^ is the control group without ferric. The first-order models (FO, FOMT, and FOFO); the Monod models (Monod and MQ); and the Gompertz models (MGM and GM) at the given ranges (0 < *k_n_* < 1) had similar kinetic behaviors and demonstrated similar dose–effect relationships between the dosages and kinetic parameters. The medium dosages (0.45–6.75 mg⋅L^−1^) demonstrated a lower rate constant than the control and higher dosages (9.0–22.5 mg⋅L^−1^), further indicating that ferric accelerated the degradation speed of poorly biodegradable organics.

The time dependency *γ* of the methane production also weakens for medium dosages, indicating the acceleration of methane production. The lag time *λ* is an essential kinetic parameter representing a relief period in ammonia inhibition, for example [27]. The lag time decreased when the dosage increased, indicating that that inhibition was mitigated [31].

### 3.3. Changing of Methanogenic Kinetic Patterns in the Batch Test

The cumulative methane production could be fitted to the multiple kinetic models (M1~M8) with the Levenberg–Marquardt algorithm (R^2^ > 0.963, rRMSE < 18.78), and the modified Gompertz model (MGM) had the lowest rRMSE (2.13%) for multiple dosages and the highest (average) fitting (R^2^ < 0.997). The cumulative methane production curve could be approximately reproduced by all the models listed (M1~M8). A visual inspection showed that the residuals for the MGM were evenly spread across the values of BMP(t) and time (Figure S1).

The first methane production peak (hour 1) was slightly overestimated by the first-order models, including FO, FOMT, and FOFO. Moreover, the period (hours 2–26) after the first methane production peak was vastly underestimated by the first-order models. A similar overestimation of the first peak and an underestimation of the following period was observed in the Monod models (Monod and MQ). The model estimation after the first peak was improved by the modified time dependency (FOMT and MQ), with an accuracy trade-off between the first peak and the following period. On the contrary, the first methane production peak was slightly underestimated by the dual exponential functions (MGM and GM). Furthermore, the period after the first methane production peak was somewhat overestimated by the dual exponential functions. The LFM performed very similarly to the MGM in terms of giving the actual values.

The second methane production peak (hour 27) trends were slightly underestimated by all models, but the period after the second methane production peak (hours 28–65) varied between models. The period was overestimated by the first-order models. A similar severe overestimation was also seen for the Monod models. Model estimation after the second peak was also improved by the modified time dependence, while the accuracy loss was smaller than for the first peak. The period was only slightly overestimated by the dual exponential functions (MGM and GM). The most stable residuals in the second methane production peak and the following period were produced from the LFM model.

The last period (hours 66–84) was different not only in terms of the model but, also, in terms of the dosage. The last period was underestimated by the first-order models. The underestimation of FO and FOFO decreased significantly when the dosages were at the medium levels Fe2.25–Fe6.75 (*p* < 0.05). The last period was underestimated even more by the Monod model, which also improved at the medium dosages. The underestimation of the last period was improved by the modified time dependency (FOMT and MQ). The last period was also underestimated by the dual exponential functions at the low and high dosages, while it was overestimated at the medium dosages. The characteristics of the last period might be due to the poorly biodegradable organics. The kinetics described the standard patterns of methane production. The differences in the last period indicate that ferric changed the kinetic patterns of the poorly biodegrading organics at the medium dosages.

The dual exponential function (exp(f[exp(time)])) demonstrated better fitting than the other mathematic forms of kinetics. A dual exponential is an interesting mathematic pattern, which may be linked to the syntrophic nature of microbial methane production. The dual exponential function (M6 to M7) grows much more quickly than an exponential function (M1~M3), which shows lower residuals after both methane production peaks. Similar residuals can also be found in previous research [12].

### 3.4. Effect of Dosage on Biochemical Methane Potential by GM-Based NMA

All the kinetic models suggested that the biochemical methane potential was maximally enhanced by Fe2.25, while suggesting quite different growth rates of 3.62–9.67%. The biochemical methane potential was enhanced by a maximum of 9.67% for Fe2.25 in the Monod model, which is the kinetic pattern in anaerobic digestion model No. 1. The first-order model and Gompertz model suggested 7.61% and 4.58% for Fe2.25, respectively. The biochemical methane potentials differ between the methane production kinetic models, which causes confusion. This confusion has limited the widespread application of kinetic models [12].

**Figure 4:**
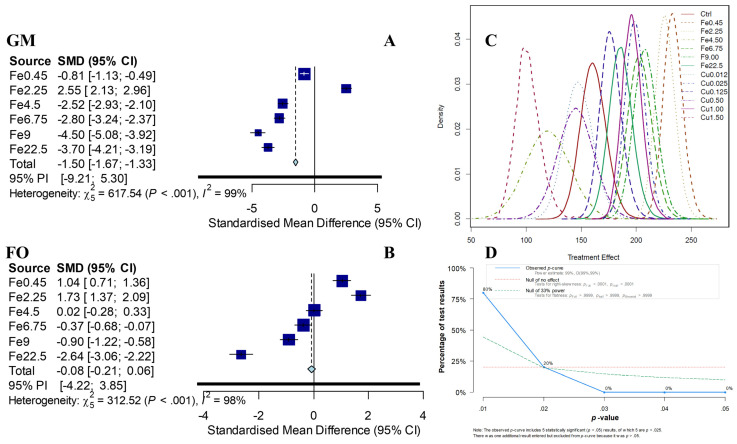
Prediction of the changing rate of the methane yield with different ferric dosages by the Bayesian network meta-analysis based on the biochemical methane potential at a 95% confidence level. (**a**) Gompertz model-based prediction, (**b**) first-order model-based prediction, (**c**) treatment effect density distribution of all dosages in the Gompertz model, and (**d**) *p*-curve. The low *p*-value and the impartial funnel plot indicated that the prediction is reliable in the reported case with acclimated microbes.

The biochemical methane potential was enhanced by a maximum of 2.55% (with a 95% confidence interval (2.13%; 2.96%)) for Fe2.25, as indicated by the network meta-analysis based on the Gompertz model (GM; Figure 4). The result (2.55%) was similar to those based on the first-order model, which was 1.73% (with a 95% confidence interval (1.37%; 2.09%)) for Fe2.25. The network meta-analysis showed significant heterogeneity (*p* < 0.001) in both the Gompertz model and the first-order model. Increasing or decreasing the ferric dosage reduced the biochemical methane potential in batch tests and the methane yield in a continuous reactor. The Bayesian network meta-analysis showed that the differences between the biochemical methane potential and cumulative methane production might differ due to the superposition of the multiple effects of ferric. Therefore, the “true” effects of the biochemical methane potential can be estimated by eliminating the superposition. The Right-Skewness tests showed a significantly right-skewed *p*-curve, indicating that there is a “true” effect [24]. The funnel plot suggests that the small-study effects are acceptable (Figure S2).

### 3.5. Changing of Methane Yield and Performance in Semicontinuous AnMBR

The paired-samples *t*-test indicated that only the ferric significantly influenced the methane yield in semicontinuous AnMBR (*p* < 0.05). The ferric stages (sFe) with an optimized dosage of Fe2.25 mg⋅L^−1^ were able improve the methane yield by an average of 2.27% ± 0.67% (days 42–84, sFe), as compared with the control stage (days 1–42, sCtrl). The average enhancement rate (2.27% ± 0.67%) was in accordance with the previous evaluation using the Bayesian network meta-analysis (2.55%) (with a 95% confidence interval (2.13%; 2.96%)), with a difference of less than 0.5%. The prediction bias of methane production from batch to continuous was reduced from 11%~19% to less than 0.5%.

**Figure 5:**
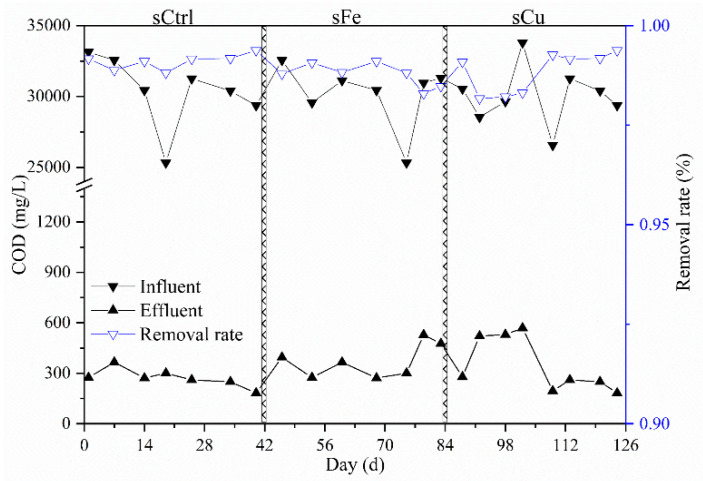
The influent and effluent COD of the anaerobic membrane bioreactor (AnMBR) without (sCtrl) augmentation and with ferric (sFe) and copper (sCu) augmentations at the Bayesian network meta-analysis-optimized dosages, respectively. The COD removal rate unexpectedly increased and met the discharging standard. Despite that, the methane yield with ferric augmentation in the long term was improved by 2.27%, just as predicted.

The paired-samples *t*-test indicated that ferric and copper significantly influenced the reactor performances in semicontinuous AnMBR (*p* < 0.01). Both ferric and copper significantly improved the organic loading rate (Table 1). The effluent COD of AnMBR was 351.69 ± 73.23 mg⋅L^−1^ in 126 days, which firmly meets the discharging standard (Figure 5). The sequencing batch reactor’s performance relied on the efficiency of each batch [19]. The ferric was able to enhance the degradation of poorly biodegradable organics by accelerating the second peak’s degradation speed and changing the last period’s kinetic patterns. The ferric therefore shortened the batch cycle, leading to a higher loading rate (12.36 ± 0.41 kg_COD_⋅kg_VSS_^−1^⋅d^−1^) than the control or copper period. On the other hand, the copper increased the methane yields by only 0.32% in the continuously operated reactor. The other effects, including the organic loading rate and effluent COD, were less stimulated by copper than ferric.

## 4. Discussion

### 4.1. Potential Mechanisms of Methanogenic Kinetic Response to Ferric

The difference between the BMP batch and the continuous-flow reactor could be attributed to a combination of mechanisms related to ferric and copper, e.g., the electron acceptor and donator. Copper has limited impacts on the methane yield. The copper could be the reciprocal regulation of the genes encoding two MMOs (Methane monooxygenase) intracellularly. Ferric has more mechanisms than copper in methanogenesis. Ferric could accept the electron and donate it later for the methanogen. Ferric could also stimulate interspecies electron transfer by many mechanisms. Ferric could decrease the distance between two groups. Ferric could conduct an electron between two groups in the syntrophic methanogenic consortia, which also could stimulate a methane yield [9]. Ferric widely participates in methanogenic biochemical processes, as in enzymes. The mechanisms of extracellular electron transfer (EET) were extensively elaborated recently, showing that Fe (II) works as an electron shuttle. Fe(II) oxidizers can perform a direct electron transfer to reduce CO_2_, which is involved in the Direct EET hypothesis [32,33]. The shuttle electrons for interspecies extracellular electron transfer move by the redox cycling of Fe (II)/Fe (III). The electron shuttle may be a more convincing argument than flocculation for the examples herein, given the enhancement for Fe2.25. Cycling also relies on the oxidation–reduction potential. Magnetite (Fe^2+^Fe_2_^3+^O_4_^2−^) is also a commonly tested conductive material that mediates the interspecies electron transfer (IET). It is known as a conductive-material-mediated IET, and more researchers tend to regard it as a type of direct IET. Direct IET is an alternative IET to interspecies hydrogen transfer or interspecies formate transfer. Magnetite could even trigger direct IET in a methanogenic sludge and could promote methane production from propionate [34].

On the other hand, iron also plays significant roles in Flavin-based electron bifurcation. Ferredoxins are iron–sulfur proteins that mediate electron transfers for strictly anaerobic bacteria and archaea. Ferredoxins act as low-potential terminal acceptors that, upon reoxidation, drive electrochemical proton pumps such as ferredoxin-NAD reductase (Rnf) [35] and ferredoxin-proton reductase [36]. Reduced ferredoxins also enable difficult reductions, such as protons to hydrogen, nitrogen to ammonia, and CO_2_ to formyl groups [37].

Understanding the dose–effect relationships between batch and continuous anaerobic digestion is a topic of great interest. Network meta-analyses may not be as powerful as they are supposed to be for long-term evaluations, given the partial limitations. However, a network meta-analysis still showed better accordance than the many methods attempted herein in terms of representing the batch and reactor processes. One of the main reasons for this was that ferric combines many effects, e.g., flocculants, enzymes, and electron transport, in methane production. The Bayesian network meta-analysis model allowed us to estimate the probability of the combined effects as a conditional probability during the processes of methane production [38].

### 4.2. Deciphering the Methanogenic Kinetic Response to Ferric

**Figure 6:**
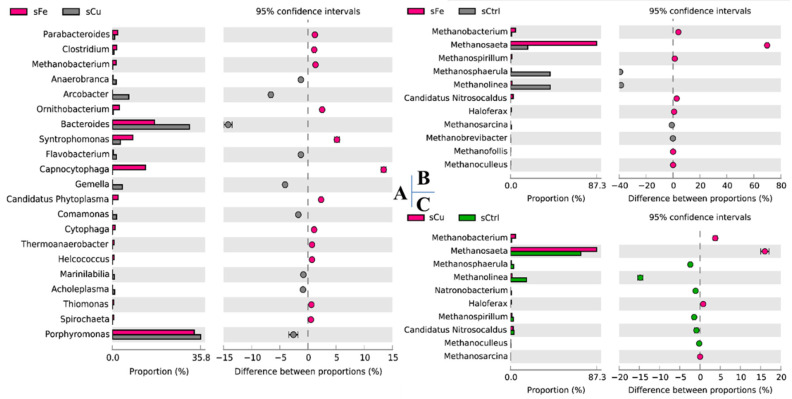
The STAMP plot comparing the microbial community of the AnMBR with long-term ferric and copper augmentation: (**A**) sFe and sCu, (**B**) sFe and sCtrl, and (**C**) sCu and sCtrl. The *Methanosaeta* is stimulated by both ferric and copper. The ferric dosage also stimulated *Flavobacterium* and *Syntrophomonas* as syntrophic methanogenic consortia, while copper inhibited them, e.g., *Bacteroides*.

The community structure results showed that *Methanosaeta* significantly increased with ferric and copper supplementations in the sFe and sCu stages (Figure 6). The triggered effects on acetoclastic *Methanosaeta* may be sustained in the copper period [39], as ferric accumulated in the reactor [40]. Kinetic patterns represent methane production in a mathematics form. Overestimation between the two peaks by first-order models and Monod kinetics implies that the methane production for that period does not follow the single logarithmic multiplication pattern. The lower residuals using the dual exponential kinetics implied two syntrophic microbes with different growth rates. Each microbe has its own time-depended growth rate. They collaborate deeply as a dual exponential function. Simultaneous syntrophism of the two groups of microbes affected the methane production. The better fit in the last three stages (after the first peak) using the dual exponential kinetics implies that syntrophic methane production played a primary role in the last three methane production periods.

The network analysis showed the ferric dosage (0.45, 22.5 mg⋅L^−1^) significantly affected the biochemical methane potential (*B_n_*) and the rate constant (*k_n_*) in the first-order and the Monod models. The results strongly implied that the ferric dosage was linked more with the first group in terms of syntrophic consortia. The ferric dosage also showed a significant correlation with the lag time. The results implied that the ferric dosage might accelerate the syntrophism [34], instead of directly linking with the second group in the syntrophism.

The kinetic patterns also implied different pathways for enhancing the methane production. For example, the overestimating of the first-order model suggested that methane production may be lower than it is supposed to be. Therefore, pretreatment, pre-acidification [18], etc. are potential countermeasures. Extending the logarithmic multiplication after the first peak may also be helpful. The better fit of the Gompertz model suggested a non-negligible lag in the system. The lag may occur due to inefficient syntrophism or even revisable inhibition. Therefore, enhancing the syntrophism by triggering a direct interspecies electron transfer (DIET) with a conductor or by increasing the aggregation with flocculation could be a solution. Ferric could play more roles as a flexible management tool [41]. The prediction of biogas production based on a better understanding of biochemical kinetics would allow for better food processing wastewater treatment to be established to decrease pollutants and increase energy recovery.

## 5. Conclusions

The effects of ferric and copper on methane production were investigated in batch and lab-scale reactors in this study. A consistent enhancement of methane production from batch (2.55% ± 0.42%) to the reactor (2.27% ± 0.67%) was achieved using the Gompertz kinetic-based Bayesian network meta-analysis. Ferric enhanced the degradation of poorly biodegradable organics primarily by accelerating the second peak’s degradation speed (*k_71_*) and decreasing the lag time (*λ_n_*), while copper accelerated the first peak’s degradation speed. Kinetic patterns revealed that the effect of ferric was primarily affected by syntrophism, but copper demonstrated limited impacts in the continuously operated reactor. The improved coupling as a result of the batch evaluation allowed for a predictable operation of a semicontinuous reactor with a higher methane yield and loading rate.

## Figures and Tables

**Table 1:** Changes of the methane yield and performance in a semicontinuous anaerobic membrane bioreactor (AnMBR). COD: chemical oxygen demand.

Control Strategy (Biogas-pH strategy)	sCtrl	sFe	sFe Rate (%)	sCu	sCu Rate (%)
Time (day)	1–42	42–84		84–126	
Organic loading rate (kg_COD_⋅kg_VSS_^−1^⋅d^−1^)	11.8 ± 0.3	12.4 ± 0.4	4.6	12.1 ± 0.6	2.1
Effluent COD (mg⋅L^−1^)	273 ± 48	374 ± 93	37	349 ± 152	28
Methane flow rate (NmL⋅h^−1^)	593 ± 351	632 ± 397	6.6	619 ± 368	4.44
Methane yield (NmL⋅gCOD_in_^−1^)	309 ± 51	316 ± 57	2.27	310 ± 62	0.32

## Data Availability

The data presented in this study are available on request from the corresponding author.

## Supplementary Materials

The following are available online at https://www.mdpi.com/2077-0375/11/2/100/s1: Figure S1. Facet plot of residual error for different methanogenic kinetic models. The flat curve indicated a stable fit throughout digestion. The digestion time was marked at three kinetic stages for to compare the performances of the different kinetic models: the first peak of daily methane production (1), the second peak of daily methane production (27), and the last period (66). Figure S2. The Funnel plot for the bias check and effects plots in the network meta-analysis. Table S1. Nomenclature. Table S2. Dose of ferric and copper for the methane production dynamic characteristics.
