# Supplementary material for: Prediction of the Long-Term Effect of Iron on Methane Yield in an Anaerobic Membrane Bioreactor Using Bayesian Network Meta-Analysis
Source: Membranes (Basel). 2021 Jan 31;11(2):100. doi: 10.3390/membranes11020100 (PMC7911906; doi:10.3390/membranes11020100)
Supplement: Supplementary file 1 [file membranes-11-00100-s001.pdf]

Supplementary Materials

Prediction of the Long-Term Effect of Iron on Methane Yield in an Anaerobic Membrane Bioreactor Using Bayesian Network Meta-Analysis

Dawei Yu [1,2,3,4], Yushuai Liang [1,2,3], Rathmalgodage Thejani Nilusha [1,2,3], Tharindu Ritigala [1,2,3] and Yuansong Wei [1,2,3,*]

[1] State Key Joint Laboratory of Environmental Simulation and Pollution Control, Research Center for Eco-Environmental Sciences, Chinese Academy of Sciences, Beijing 100085, China; dwyu@rcees.ac.cn (D.Y.); lys15122117710@163.com (Y.L.); nthejani@yahoo.com (R.T.N.); tharindu_st@rcees.ac.cn (T.R.)
[2] Department of Water Pollution Control Technology, Research Center for Eco-Environmental Sciences, Chinese Academy of Sciences, Beijing 100085, China
[3] University of Chinese Academy of Sciences, Beijing 100049, China
[4] BIOMATH, Department of Data Analysis and Mathematical Modelling, Ghent University, Coupure links 653, 9000 Ghent, Belgium
* Correspondence: yswei@rcees.ac.cn; Tel.: +86-010-6284-9109

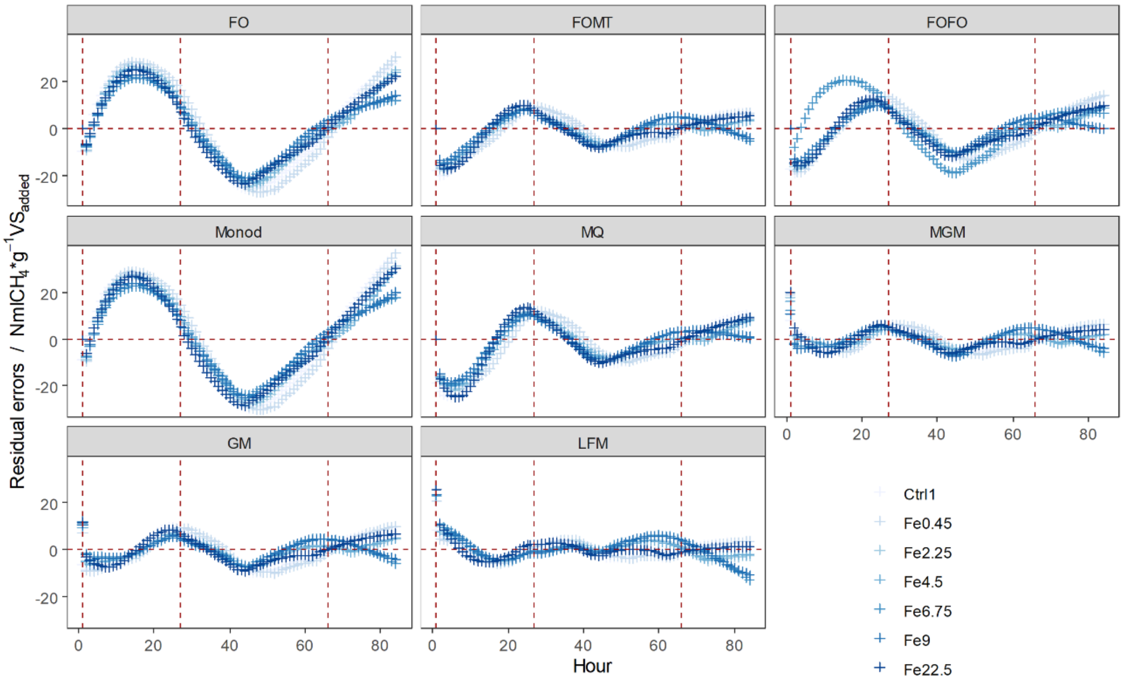

Figure S1. Facet plot of residual error for different methanogenic kinetic models. The flat curve indicated a stable fit throughout digestion. The digestion time was marked at three kinetic stages to compare the performance of different kinetic models: the first peak of daily methane production (1), the second peak of daily methane production (27), and the last period (66).

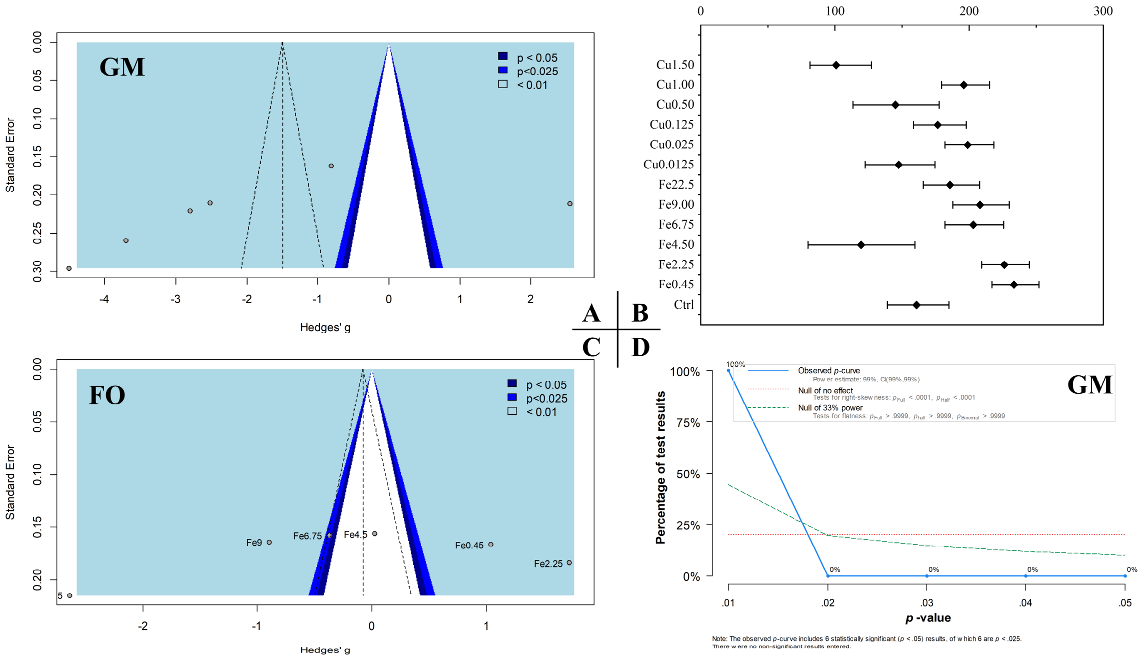

Figure S2. The Funnel plot for the bias check and effects plots in the network meta-analysis. (**A**) funnel plot of GM based prediction; (**B**) effects plot of GM based prediction; (**C**) funnel plot of FO base prediction; (**D**) percentage of test results of GM based prediction.

Table S1. Nomenclature

Symbol	Parameter	Unit
B_1	BMP of first-order rate model	$Nml \cdot gCOD^{-1}$
B_2	BMP of first-order rate model with variable order of time dependency	$Nml \cdot gCOD^{-1}$
B_3	BMP of combination of two first-order rate models	$Nml \cdot gCOD^{-1}$
B_4	BMP of Monod model	$Nml \cdot gCOD^{-1}$
B_5	BMP of quadratic Monod model	$Nml \cdot gCOD^{-1}$
B_6	BMP of modified Gompertz model	$Nml \cdot gCOD^{-1}$
k_1	rate constant of first-order rate model	1/day
k_2	rate constant of first-order rate model with variable order of time dependency	1/day
k_{31}	rate constant of combination of two first-order rate models	1/day
k_{32}	rate constant of combination of two first-order rate models	1/day
k_4	rate constant of Monod model	1/day
k_{51}	rate constant of quadratic Monod model	1/day
k_{52}	rate constant of quadratic Monod model	1/day
k_{61}	rate constant of modified Gompertz model	1/day
k_{62}	rate constant of modified Gompertz model	1/day
t	Digestion time	1/day

γ	order of the time dependence for first-order rate model with variable order of time dependency	-
X	fraction of readily degradable material for two first-order rate models	-
θ_1	lag period of modified Gompertz model	1/day
θ_2	lag period of modified Gompertz model	1/day

Table S2. Dose of ferric and copper for methane production dynamic characteristics.

**Trace Elements**	**Concentration / mg·L^{-1*}**					
Ferric (Fe^{3+})	Fe0.45	Fe2.25	Fe4.50	Fe6.75	Fe9.00	Fe22.5
Copper (Cu^{2+})	Cu0.0125	Cu0.025	Cu0.125	Cu0.50	Cu1.00	Cu1.50
